# Oil Extraction from Hemp Plant as a Potential Source of Cannabidiol for Healthy Protein Foods

**DOI:** 10.3390/antiox12111950

**Published:** 2023-11-01

**Authors:** Olga Mileti, Noemi Baldino, Mario F. O. Paleologo, Francesca R. Lupi, Maria Marra, Domenico Iacopetta, Domenico Gabriele

**Affiliations:** 1 Department of Information, Modeling, Electronics and System Engineering (D.I.M.E.S.), University of Calabria, Via P. Bucci, Cubo 39C, 87036 Arcavacata di Rende, Cosenza, Italy; o.mileti@dimes.unical.it (O.M.); oraldo.paleologo@unical.it (M.F.O.P.); francesca.lupi@unical.it (F.R.L.); d.gabriele@unical.it (D.G.); 2 Department of Pharmacy, Health and Nutritional Sciences, University of Calabria, Via P. Bucci, 87036 Arcavacata di Rende, Cosenza, Italydomenico.iacopetta@unical.it (D.I.)

**Keywords:** *Cannabis sativa* L., hemp oil, hemp flowers, hemp stalks, rheology, protein dough

## Abstract

In recent years, the increasing demand for alternative foods has shifted research toward new sources enriched with nutraceutical molecules. It is well known that many diseases are caused by oxidative stress; thus, the supplementation of antioxidants has been proposed to reduce it. *Cannabis sativa* L. is an interesting species that could provide an alternative source of antioxidants. This work aimed to investigate the possibility of optimizing the yield of cannabidiol (CBD) and recovering it from residual biomass (stalks), valorizing the residual biomass, and using this for protein bar preparation. Different extraction methods were used, and High-Pressure Liquid Chromatography (HPLC) analysis was used to analyze the extracts. Antioxidant power was investigated using the 2,2-Diphenyl-1-picrylhydrazyl (DPPH) and 2,2′-azinobis-3-ethylbenzothiazoline-6-sulfonic acid (ABTS) assays. The best results in terms of CBD yield were obtained via dynamic maceration after decarboxylation with a quantity of 26.7 ± 2 mg_CBD_/g_raw material_ from inflorescences. The extract also shows good antioxidant power with an IC50 value of 38.1 ± 1.1 µg/mL measured using the DPPH assay. The CBD extract was added to the hemp oil to obtain dough for protein bars. The doughs were studied by taking rheological and technological measurements, and it was found that the protein bars could provide an excellent means for the consumption of products enriched with antioxidants because their CBD anti-inflammatory activity is preserved after cooking.

## 1. Introduction 

In recent years, the increasing demand for alternative foods has shifted the research toward new kinds of nutrients. One of the most important new scientific trends in food research involves cannabinoids, which are naturally present in *Cannabis sativa* L., a widely diffused dioecious plant belonging to the Cannabaceae family. In the past, it was extensively used as a drug and a source of textile fiber [1]. The genus Cannabis is divided into three classes: the first, named *C. sativa* L. and commonly known as hemp, is rich in fiber; the second, *C. indica* Lam., is the drug-type, containing high levels of the narcotic compound Δ9-tetrahydrocannabinol (Δ9-THC); the third, *C. ruderalis* Janish, shows intermediate properties that are intermediate between the first two [2]. While the drug-type class has been thoroughly investigated for a long time thanks to its pharmaceutical properties, scientific researchers are now focusing on the fiber-type class, both for food and pharmaceutical purposes [2,3]. Moreover, hemp seeds are widely used in foods to extract high-nutritional-value oil, which is rich in polyunsaturated fatty acids [3], and hemp inflorescences are also characterized by a wide range of chemical species, among which the most interesting are cannabinoids [2]. Cannabinoids can be used as supplements in foods and not only for therapeutic purposes. They belong to the terpenophenolics class, being generated by the alkylation of an alkyl resorcinol and a monoterpene oligomeric unit. The main fraction of cannabinoids in the fiber-type is composed of cannabidiolic acid (CBDA) and cannabigeloric acid (CBGA), with their decarboxylated types, cannabidiol (CBD) and cannabigerol (CBG), respectively [2,4]. 

CBD is a very important and well-known molecule because of its antioxidant, anti-inflammatory, and antibiotic activity, as well as its anxiolytic and neuroprotective properties [5,6]. The extraction method chosen to extract CBD is based on the raw material source and the desired grade of selectivity. CBD-enriched extracts can be obtained by employing ethanol, which is the preferred solvent thanks to its selectivity and the easy separation method necessary to obtain CBD extracts, but it is characterized by its poor yield [2,7]. Yields can be improved by raising the extraction time or temperature, although long-term exposure time to high temperatures could degrade thermolabile cannabinoids [7]. Another technique used to obtain CBD is Soxhlet extraction, in which solvent reflux guarantees the maximum extraction of phytochemicals, despite the fact that it can compromise the integrity of the cannabinoids extracted [7]. 

To obtain CBD-enriched extracts, a decarboxylation stage could be inserted before the extraction process, to promote the conversion of CBDA into CBD [8,9]. In recent years, CO_2_ supercritical fluid extraction has been investigated as a green alternative to classic solvent extraction [10]. The extraction yield of this method depends on the operative condition, pressure, and temperature. In particular, yield increases with pressure, along with a decrease in extraction time [3]. In some cases, 5% *v*/*v* ethanol is added to CO_2_ to improve the extraction of cannabinoids, while it has been demonstrated that using only CO_2_ increases the CBD purity in the extracts [11]. 

The properties of CBD oil from inflorescences and hemp seed oil have recently encouraged studies on food products based on hemp oil and protein, in which the positive effects of hemp protein are accompanied by the antioxidant and anti-inflammatory properties of CBD-rich hemp oil [3,12]. 

In this paper, several methods for the extraction of CBD and its precursor, CBDA, from hemp inflorescences and stalks are investigated. The extracts were analyzed via the HPLC method and their antioxidant power was tested. The CBD extract was used to enrich the hemp oil obtained from protein flour, conferring it a high antioxidant power. The oil was used in dough formulation.

Thus, the objectives of the present study are to evaluate the possibility of obtaining CBD from different parts of a hemp plant for use in the formulation of rheologically optimized doughs for protein bars and show that baked goods could be used as a great way to avoid exposure to direct heat and make use of the many benefits of CBD.

## 2. Materials and Methods

### 2.1. Raw Materials 

Flowers and stalks were manually divided from the plant to obtain hemp inflorescences (HI) and stalks (HS). 

The hemp plant, supplied by Le Querce S.r.l (Montalto Uffugo, CS, Italy), was collected from the experimental cultivation of hemp Futura 75 (carried out at Spezzano Sila, Cosenza, Italy). Futura 75 was certified with a content of Δ9-THC below 0.3% (*w*/*w*), while hemp protein flour (HPF) was kindly provided by Eco Officina Agraria S.r.l. (Arezzo, Italy). The HPF was composed of fats 24% *w*/*w*, carbohydrates 9% *w*/*w*, proteins 57% *w*/*w*, and moisture 10% *w*/*w*.

For the extraction process, only absolute ethanol (VWR Chemicals, Rosny-sous-Bois, France) was used as a solvent. Protein doughs were prepared using HPF; Hi-Maize^®^ 260 (Ingredion, Westchester, IL, USA), which is a resistant starch; Greek yogurt (Fage, Luxembourg); and tap water. CO_2_ (purity > 99.99%) was supplied by SIAD Spa (Bergamo, Italy) for supercritical extraction.

### 2.2. Extraction Methods

For antioxidant recovery, different methods of extraction were employed: supercritical fluid extraction (SFE), using CO_2_ as a supercritical fluid; Soxhlet (SE); and dynamic maceration (DME), using ethanol as solvent. 

The extractions were performed on hemp inflorescences (HI), stalks (HS), and proteic flour (HPF), using the raw material after manual separation without further treatments. After a decarboxylation process, conducted at 140 °C for 30 min in a ventilated oven (FD 53, BINDER GmbH, Tuttlingen, Germany), according to the literature procedure [8], the DHI, DHS, and DHPF fractions were obtained.

Regarding SE, 15 ± 1 g of sample and 400 mL of ethanol were used. The samples were placed into a thimble filter and located in the middle of the Soxhlet extractor. The extraction process was carried out at 78 °C for 8 h, after which the ethanol hemp-enriched extract was collected [13].

In order to perform the DME, 1 g of sample was used, added to 40 mL of ethanol, using the solvent/sample ratio suggested by literature [2]. The DME process was carried out at room temperature for 15 min under magnetic stirring. Afterwards, the ethanol was recovered and the exhaust sample was extracted again, using the same procedure, for a second time. The obtained extract was collected and the exhaust hemp sample was used for a third cycle, adding 20 mL of ethanol, and obtaining a final extract amount of 100 mL. 

The extracts were isolated by means of a Rotavapor system (Heidolph G3, Hei-VAP Value, Heidolph Instruments, Wood Dale, IL, USA), and the yield was calculated by weighting. Extractions were performed in triplicate and the maximum yield was calculated according to the following equation:

(1) $${Yield}_{max}=\frac{mass\;of\;extracted\;(mg)}{mass\;of\;\mathrm{feed}\;(g)}$$

Determinations were conducted in triplicate.

Finally, 25 ± 1 g of grounded samples were used for SFE, carried out in a laboratory-scale plant (Spe-ed SFE, Applied Separations, Allentown, PA, USA) at 40 °C and 360 bar. A first phase of 30 min, in a static mode, followed by dynamic phases of 15 min were adopted for SFE. The static phase of 15 min was interposed between two dynamic phases, with a total extraction time of 140 min. The obtained extracts were collected in a volumetric flask, weighed, and the maximum yield was calculated according to Equation (1).

Soxhlet extraction was performed with 15 ± 1 g of HPF and 400 mL of analytical grade n-hexane (Sigma-Aldrich Co., St. Louis, MO, USA). After extraction, the obtained oil was isolated through a Rotavapor at 50 °C (Heidolph G3, Hei-VAP Value, Heidolph Instruments, Wood Dale, IL, USA), and the yield was calculated by the Equation (1).

### 2.3. Proteic Dough Preparation

In this study, a high-protein bar formulation was studied as a model, using proteins from the hemp plant, which has a high nutritional value [14,15]; Greek yogurt; resistant starch; and CBD oil, as a source of antioxidants. According to [16], a base formulation (S1), having 28% *w*/*w* on the dry product of hemp protein, without Greek yogurt was studied. The model bars were obtained by adding a resistant maize starch, rich in amylose, with a low glycemic index [15]. Starting from an initial formulation, the fresh Greek yogurt was added, at different percentages, because of its ability to improve the final dough consistency and quality, resulting in a softer system [17]. 

Four formulations, with increasing amounts of Greek yogurt, were studied. The ingredients’ percentages are reported in Table 1. A total 1% *w*/*w* of CBD oil extracted from inflorescences was added to the dough of all samples. 

High-protein bars were made by mixing all the ingredients by means of a Brabender Farinograh (Belotti Instrument, Peschiera Borromeo (MI), Italy). The velocity was set at 63 rpm and the temperature at 24 °C. The mixing time for all samples was 20 min.

At the end of the mixing stage, part of the dough was bar-shaped and cooked in the oven, and another part underwent rheological measurements.

### 2.4. HPLC Analysis

CBD and CBDA content in the extracts were evaluated using a Smartline HPLC system (Knauer, Berlin, Germany) consisting of a degasser, a pump, and a UV detector 2600. Chromatographic separation was accomplished using a 150 mm × 2 mm i.d. C18 Ascentis, with a precolumn (Supelco, Darmstadt, Germany). The column temperature was set at 32 °C. The mobile phase was composed of 0.1% *v*/*v* formic acid in acetonitrile (A) and 0.1% *v*/*v* formic acid in water (B) at a flow rate of 0.1 mL/min. In Brighenti et al.’s study [2], during analysis, the gradient elution was varied as follows: 0–13 min, 60% A; 13–17 min, from 60 to 80% A; 17–22 min, from 80% to 90% A, which was maintained for eight minutes. Finally, a 15-min post-running time was imposed. This method is validated for quality control of pharmaceutical products, according to the ICH guidelines [2].

Absorbance spectra were recorded every 1 s, between 200 and 450 nm, with a bandwidth of 8 nm. Chromatograms were acquired at 210, 220, 235, and 275 nm. The measurements were performed in triplicate. For both CBD and CBDA, a calibration curve was obtained by performing HPLC analysis on different CBD and CBDA standard solutions, at concentrations of 0.01, 0.025, 0.1, 0.5, and 1 mg/mL (Sigma-Aldrich Co., St. Louis, MO, USA). Before injection into the HPLC system, the extracts were filtered using a 0.45 μm PTFE filter.

### 2.5. Antioxidant Activity

The antioxidant activity of extracts was investigated by two methods: 2,2-Diphenyl-1-picrylhydrazyl (DPPH) assay and 2,2-Diphenyl-1-picrylhydrazyl (DPPH) assay. Firstly, the radical scavenging properties of the extracts towards the 1,1-diphenyl-2-picryl-hydrazil (DPPH) radical were investigated as described by Iacopetta et al. [18], with some modifications. Briefly, 20 μL of each extract, properly dissolved in ethanol, were mixed with 180 μL of a 0.1 mM DPPH solution in methanol, in a 48-well plate, to obtain seven different concentrations (from 100 to 5000 µg/mL). As a negative control, 20 μL of ethanol in 180 μL of DPPH methanol solution was diluted. The mixture was then shaken and incubated at room temperature, for 30 min, protected from the light. The scavenging activity was determined at 517 nm using a microplate reader. 

The radical scavenging properties of the extracts against the 2,2′-azino-bis(3-ethylbenzothiazoline-6-sulfonate) radical cation (ABTS^•+^) were extrapolated as described by Iacopetta et al. [18], with minor adjustments: 2 mM ABTS and 70 mM potassium persulfate water solutions were mixed, incubated for 16 h at room temperature, and protected from light to obtain the ABTS^•+^ radical stock solution. The latter was ethanol-diluted until the absorbance at 730 nm was 0.70 ± 0.02. Before use, 2 μL of each extract, dissolved in ethanol, was added to 198 μL of the ABTS^•+^ solution in a 48-well plate to obtain seven increasing concentrations (from 100 to 2000 µg/mL). After being shaken and incubated for 5 min at room temperature (in the dark), the scavenging activity was determined at 730 nm. Also, in this case, a negative control was inserted (2 μL of ethanol and 198 μL of the ABTS^•+^ solution). For both methods, the radical scavenging activity was expressed as inhibition percentage (%*I_i_*) of each extract compared with the respective control, according to the following equation:

(2) $${\%I}_{i}=\frac{A_{0}-A}{A_{0}}\cdot 100$$

where the subscript *i* refers to DPPH and ABTS, with respect to each assay; *A*_0_ is the control reaction absorbance; and *A* is the absorbance in the presence of samples. IC_50_ values determination was obtained from the IDPPH and IABTS percentages, using GraphPad Prism 9 software (GraphPad Inc., San Diego, CA, USA). As a positive control, Trolox was employed. All experiments were performed three times, each in triplicate. Data are expressed as mean values ± standard deviation (SD).

### 2.6. Rheological Characterization 

The doughs were characterized by rheological measurements performing tests in dynamic mode. Frequency sweep tests were carried out at 20 °C, 40 °C and 60 °C in the linearity region, in a range of 0.1–10 Hz, preliminarily evaluated by stress sweep and time sweep tests. Furthermore, temperature ramp tests in heating mode were performed from 20 °C to 100 °C, at 1 °C/min, and at 1 Hz, in linearity. All rheological tests were carried out by means of a rotational rheometer (HAAKE MARS III, Thermo Fisher Scientific, Braunschweig, Germany) equipped with a parallel plate geometry (diameter = 20 mm; gap = 2 mm ± 0.1 mm) and a Peltier system for controlling the temperature. All measurements were conducted twice. To avoid water evaporation phenomena during tests, silicone oil of 120 cSt (VWR Chemicals, Briare, France) was used.

The frequency sweep tests were interpreted using the weak gel model [19]:

(3) $$G^{*}=A\omega^{\frac{1}{z}}$$

where G* is the complex modulus, ω is the frequency, *A* is a measure of gel strength, and *z* is a measure of gel structuration.

### 2.7. Baking Trials and Characterization 

The formulations, studied by rheological techniques, were baked in a ventilated oven (Unox XF013, Stefania, Padova, Italy) for 30 min at 180 °C, and then for 15 min at 140 °C.

The snack bars were further characterized post-baking by color measurements performed through a colorimeter (Croma Meter CR-400, Ramsey, NJ 07446, USA) in the CieLab color space. The colors were defined by color coordinates in CieLab Space: L*, the axis of lightness; a*, the axis of red/green transition; and b*, the axis of yellow/blue transition. 

According to Gonzales, it is possible to evaluate the browning index (*BI*) [20]:

(4) $$BI=\frac{100\;\cdot \left(x-0.31\right)}{0.172}$$

where

(5) $$x=\frac{X}{X+Y+Z}$$

where *X*, *Y*, and *Z* are the tristimulus values, evaluated in the CIE XYZ color space, based on the three-component theory of color vision (red, green, and blue) [21]. A preliminary calibration was conducted before the measurements. 

After baking, CBD content in the protein snacks was extracted by maceration in ethanol for 12 h (1 g sample in 50 mL ethanol) and quantified through HPLC methods, as reported in Section 2.4.

## 3. Results and Discussion

### 3.1. Extraction and Characterization of Extracts

The extraction was performed by means of three different methods: Soxhlet extraction, dynamic maceration, and supercritical fluid extraction. The first and second methods have been used for many years, even though dynamic maceration is an improvement of classic maceration. The SFE method was used because it is a good green technology and produces solvent-free extracts, contrary to SE and DME, for which a subsequent separation phase in a rotavapor is needed. Moreover, as CBD is a thermolabile molecule, the SFE can guarantee a low extraction temperature [7], while the adopted high pressure increases the recovery of polar substances [3]. The extraction from the different parts of hemp was optimized to obtain the maximum yield. Finally, the different techniques were compared. Solvent selection for Soxhlet and dynamic extractions was evaluated according to the literature in order to obtain an oil rich in CBD and CBDA from flour, flowers, and stalks. 

The solvent extraction processes were conducted using ethanol, which is considered the optimal solvent for the extraction of polar molecules, such as cannabinoids [2]. The obtained data from the extraction are shown in Table 2, where it is possible to see that no appreciable amount of extract was obtained from the stalks using the dynamic maceration. In contrast, the extraction from inflorescences produced appreciable amounts of substances, as already reported by other authors [11]. The yield of Soxhlet extraction is the highest of all samples, compared with DME and SFE techniques, with the exception of HPF and DHPF (Table 2). The processing of HPF gave low yields, because of the flour cohesivity, and the stirring and pressure applied during the DME and SFE process, which favor the contact between the solid matrix and the extracting fluid. Concerning stalks, it is only possible to obtain an extract by SE, probably because of the higher temperature used compared with dynamic extraction. The obtained results confirm the critical role played by temperature, as already reported by Brighenti et al. [2], who analyzed different extraction processes.

Finally, the oil yield was quantified and checked for the presence of polar substances, without using any co-solvent, as reported by [2]. The obtained data show that 75% *w*/*w* of the amount of total fat is recoverable from HPF thanks to the SFE, confirming a good affinity of CO_2_ for the lipophilic substances. This result is interesting in comparison with the maximum amount of oil extracted by the Soxhlet methodology. A maximum oil yield of 88% is obtained using hexane as a solvent, similar to the data from [3].

All the samples were analyzed by means of HPLC to identify the CBD and CBDA species extracted from the hemp plant. Before the extraction analysis, standard solutions of CBDA and CBD were examined and the calibration curves were generated to identify and quantify the cannabinoids. According to the literature [2], CBDA and CBD are distinguishable because they have different UV spectra. In particular, the CBDA spectrum shows three different absorption maxima, with the stronger being at 220–223 nm, the second one at 266–270 nm, and the weaker at 305 nm. On the other hand, the CBD spectrum is characterized by two absorption maxima: the first at around 210–215 nm and the second at 270 nm. Table 3 reports the results obtained from HPLC characterization, in terms of CBDA and CBD components from HI and PF. 

For all the investigated samples, HPLC analysis shows that the decarboxylation process increases the CBD quantity and decreases the CBDA concentration. In addition, the results show that dynamic maceration is the optimal extraction technique for maximizing CBD yield, as reported by Brighenti [2]. Soxhlet extraction, despite being an exhaustive extraction, probably causes the degradation of cannabinoids because of the high temperature necessary and the long extraction times involved. Indeed, the obtained values of CBD and CBDA were lower than those obtained by the other techniques [22]. As expected, the non-polarity of the adopted supercritical fluid generally lowers the amount of extracted cannabinoids compared to ethanol-based methods. Even if the hemp oil yield from HPF is higher, the richness in cannabinoids is lower compared with the oil extracted from HI. The obtained values are in line with the literature data [22,23]. Finally, the stalks analysis revealed the presence of CBD in higher concentrations than CBDA, already in their native state. HI and HPF decarboxylation increases the CBD amount, making them interesting by-products that are useful for CBD recovery from the hemp plant [24]. This result is in agreement with data from the literature, where the use of stalks for the CBD-rich oil recovery used as biofuels is shown [25,26].

### 3.2. Antioxidant Activity Results

To date, different studies have demonstrated that many compounds extracted from natural matrices possess interesting antioxidant activity [27]. It is known that oxidative stress, characterized by a higher presence of Reactive Oxygen Species (ROS), can induce dramatic effects by damaging important intracellular structures, including DNA, proteins, and lipids [28]. These effects can be prevented by employing compounds with antioxidant properties, which can scavenge ROS and other radicals. Since the investigation of the antioxidant activity is influenced by several variables, different, proper assays are required for assessing the antioxidant effects of an extract [29]. In this study, two in vitro assays, namely, ABTS and DPPH, were employed to evaluate the free-radical scavenging ability of the extracts (for further details, see the Section 2.5). As a positive control, a water-soluble analogue of vitamin E (Trolox) was adopted. The calculated IC_50_ values, expressed in µM, are reported in Table 4. First, the best ability in scavenging the ABTS radical was exhibited by DHI and HI, showing IC_50_ values of 38.1 ± 1.1 and 85.3 ± 2.4 µg/mL, respectively, which are about two- and five-fold higher than that of Trolox (15.9 ± 1.3 µg/mL). 

The result, related to the extract obtained with the DM methodology, is in agreement with the extracted CBD values evaluated by HPLC investigations, which are the highest among the samples subjected to antioxidant assays. A similar activity was detected for DHS (IC_50_ = 87.2 ± 2.2 µg/mL), whereas a lower one was found for HS (IC_50_ = 125.1 ± 3.3 µg/mL). In both cases, Soxhlet extraction from stalks leads to lower CBD amounts and, therefore, a lower antioxidant capacity of the extracts. Finally, HPF and DHPF, extracted by the SFE process, were not able to scavenge the ABTS radical (IC_50_ > 2000 µg/mL). According to HPLC analyses, all the decarboxylated samples, possessing higher amounts of CBD, show higher antioxidant activity than the untreated samples.

A different behavior was recorded for the ability to scavenge the DPPH radical. Indeed, the best scavenging ability was found for DHS (IC_50_ = 348.5 ± 2.4 µg/mL), followed by DHI and HS, whose values were very similar (IC_50_ of 535.7 ± 4.7 and 550.1 ± 4.5 µg/mL, respectively). Again, HFP and DHFP were unable to scavenge the DPPH radical (IC_50_ > 5000 µg/mL). In conclusion, the extracts demonstrated better activity in inhibiting the radical ABTS compared with DPPH, in agreement with [30].

### 3.3. Rheology

In light of the good yield of hemp oil from PF and the high quantity of antioxidants, a possible way to introduce bioactive antioxidant compounds in foods is to enrich the hemp oil, from seeds, with the extract from the inflorescences. Doughs were prepared using PF, to acquire formulations suitable for the preparation of protein bars (25% protein, dry basis); resistant starch (Hi-Maize), to improve the dough structure [15]; and 5% *w/w* of fat basis, from HI oil obtained by means of DME, to enrich the bars in CBD. All samples were characterized as reported in the materials and methods section.

**Figure 1:**
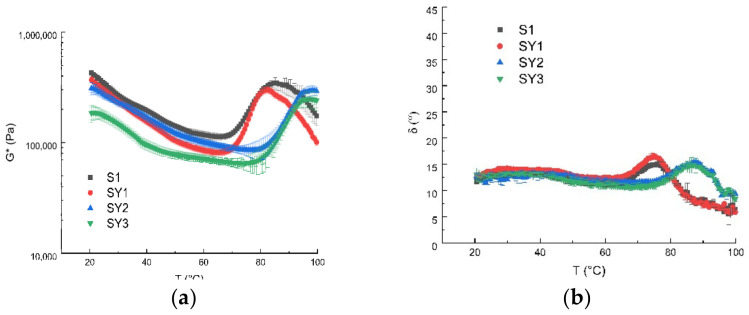
Time cure of samples in terms of G* (**a**) and phase angle (**b**), where S1 is the base formulation without Greek yogurt and the others with Greek yogurt added, at the percentages reported in Table 1.

For all samples, the temperature ramp test showed the complex modulus evolution that decreases with increasing temperature. For starch-based systems, the typical trend of the complex modulus shows a first G* decay, corresponding to an increase in molecular mobility caused by the increasing temperature, followed by a quite sharp increase of G*. The latter increase is due to the gelatinization phenomena that modify the material’s microstructure [3]. The possible accentuated growth of the complex module depends on the nature and origin of the flour, as well as on the quantity of water, and so on. The doughs, obtained with or without the addition of Greek yogurt, showed an increase in G* after 60 °C. S1 and SY1 showed a similar behavior and gelatinization temperature, in agreement with other literature formulations [31], while samples richer in Greek yogurt showed a shift of the G* curve toward higher values of gelatinization temperature. The yogurt proteins probably have a competitive effect against resistant starch in the gelatinization process. Moreover, the angle phase values (Figure 1b) suggest a solid-like behavior for all samples in the investigated temperature range, with values ranging between 5° and 25°. 

**Figure 2:**
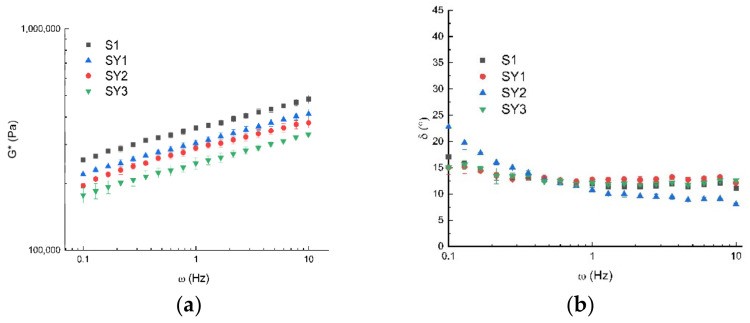
Frequency sweep test of samples at 20 °C in terms of G* (**a**) and phase angle (**b**), where S1 is the base formulation without Greek yogurt and the others with Greek yogurt added, at the percentages reported in Table 1.

Figure 2 shows the G* and angle phase values at different frequencies and at 20 °C. It is possible to see that the systems are solid-like because δ is lower than 45°. The consistency of the dough decreases (G* lowers) with the increase in Greek yogurt quantity, even if the structure does not change too much, as shown by δ’ s trend (Figure 2b). The same trend was observed for frequency sweep tests performed at 40 and 60 °C; all data were interpreted by the weak gel model [19], the parameters of which are reported in Table 5.

From the data reported in Table 5, it can be observed that, for each temperature, the A parameter decreases from S1 to SY3, indicating that the addition of Greek yogurt has the effect of weakening the sample without changing the structure, as shown by the *z* values. On the other hand, for each sample, the strength of the structure decreases with the increase in temperature; also, in this case, the z parameter is almost constant with the temperature.

### 3.4. Application

**Figure 3:**
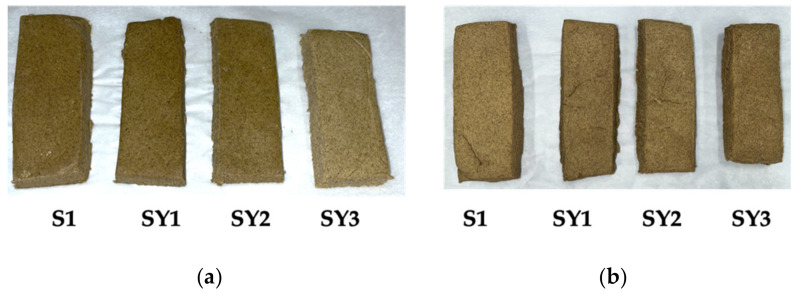
Protein snack pictures taken before (**a**) and after (**b**) cooking, where S1 is the base formulation without Greek yogurt and the others with Greek yogurt added, at the percentages reported in Table 1.

The formulations studied in the rheological analysis were used for the preparation of snack bars. Figure 3 shows the pictures taken before (a) and after (b) baking the different studied doughs.

In particular, observing Figure 3a, the increasing amount of yogurt in the dough gradually leads to a lighter color of the snack bars, becoming more evident in the SY3 formulation. After baking (Figure 3b), the difference in the coloring of the four formulations is markedly less visible, with similar values of color parameters. L*, a*, and b* are reported in Table 6. In fact, samples show similar values, but the browning index reveals an effect in the samples prepared with yogurt. Indeed, the BI parameter increases with the increase in yogurt, suggesting that, after cooking, the latter is responsible for a more pronounced browning. This effect, in agreement with literature results [32], is related to Maillard reactions that occur in cooking due to the content of sugars. In particular, although Greek yogurt contains low amounts of lactose [33], the latter is involved in Maillard reactions and promotes the browning of the samples [32].

The presence of CBD in the cooked dough was verified and quantified through HPLC analysis. In particular, a concentration of 0.36 ± 0.01 mg_CBD_/g_snack_ was found. The obtained value is in line with the CBD concentration extracted from the protein flour (as reported in Table 1). This result suggests that the incorporation of antioxidants into the formulation can preserve their function and increase the added value of the food.

## 4. Conclusions

In this study, the extraction of CBD from different parts of the *Cannabis sativa* L. plant was investigated and the CBD extracts were used in the formulation of rheologically optimized doughs for protein bar production.

From the extraction processes investigation, DME was revealed to be the most efficient method to maximize CBD yield. In particular, the decarboxylated samples and the inflorescences show a high CBD content. The extracts obtained with higher amounts of CBD demonstrated a good antioxidant power, with a higher ability to scavenge the ABTS radical compared with the DPPH one.

From the rheological characterization, doughs prepared with protein hemp flour and Greek yogurt, with the addition of CBD-enriched oil, show a solid-like behavior, with good mechanical strength and gelatinization temperatures around 80 °C, related to the presence of the resistant starch. The Greek yogurt addition to the formulations reduces the consistency of the samples and shifts the gelatinization point to higher temperatures. The hemp-based bars were also baked to obtain a finished product, which was found to preserve the antioxidant properties after baking. 

Resuming, this research highlights the possibility of recovering CBD from hemp, inflorescences, and stalks for its use in the food industry, particularly for the production of high-protein bars. However, since the mechanical and sensorial properties have not yet been studied in this work, the link between the structure and acceptability by the consumer deserves to be fully explored, possibly by adding other ingredients to the formulation, such as dried fruit/chocolate and so on.

## Figures and Tables

**Table 1:** Protein snack formulations.

	HPF % *w*/*w*	Hi-Maize^®^ % *w*/*w*	Greek Yogurt % *w*/*w*	Water % *w*/*w*
S1 *	27	35	-	38
SY1 **	27	35	3	35
SY2 **	27	35	6	32
SY3 **	27	35	9	29

* Base formulation without Greek yogurt. ** Base formulation with the addition of Greek yogurt at the percentage reported in the table.

**Table 2:** Yield (mg/g _raw material_) of extraction processes.

	HI ^1a^ (mg/g)	DHI ^1b^ (mg/g)	HS ^2a^ (mg/g)	DHS ^2b^ (mg/g)	HPF ^3a^ (mg/g)	DHPF ^3b^ (mg/g)
DME *	18.6 ± 0.9	11.7 ± 0.4	negligible	negligible	206.3 ± 10.0	180 ± 10.0
SE **	53.0 ± 8.0	52.7 ± 1.6	37.6 ± 3	35.0 ± 3.0	70.0 ± 4.5	50.0 ± 3.0
SFE ***	27.6 ± 2.0	33.7 ± 4.0	4.3 ± 0.3	5.6 ± 0.4	180.0 ± 10.0	160.0 ± 20.0

* Dynamic maceration; ** Soxhlet extraction; *** supercritical fluid extraction with CO_2_; ^1a^ hemp inflorescences; ^1b^ decarboxylated hemp inflorescences; ^2a^ hemp stalks; ^2b^ decarboxylated hemp stalks; ^3a^ hemp protein flour; ^3b^ decarboxylated hemp protein flour.

**Table 3:** HPLC results for tested samples in terms of CBD (mg_CBD/_g_raw material_) and CBDA (mg_CBDA/_g_raw material_).

		HI ^1a^ (mg/g)	DHI ^1b^ (mg/g)	HS ^2a^ (mg/g)	DHS ^2b^ (mg/g)	HPF ^3a^ (mg/g)	DHPF ^3b^ (mg/g)
DME *	CBD	13.9 ± 1.7	26.7 ± 2	0.27 ± 0.01	1.9 ± 0.1	negligible	negligible
	CBDA	35.3 ± 1.0	6.60 ± 0.50	1.4 ± 0.1	0.5 ± 0.01	negligible	negligible
SE **	CBD	5.24 ± 0.3	11.3 ± 0.1	1.90 ± 0.20	2.6 ± 0.2	0.36 ± 0.02	0.67 ± 0.04
	CBDA	10.9 ± 0.5	0.18 ± 0.01	3.40 ± 0.70	1.08 ± 0.1	0.69 ± 0.01	negligible
SFE ***	CBD	5.44 ± 0.5	14.6 ± 2	0.204 ± 0.02	0.39 ± 0.01	0.26 ± 0.03	0.75 ± 0.01
	CBDA	4.5 ± 0.2	negligible	0.573 ± 0.05	0.06 ± 0.01	0.55 ± 0.08	negligible

* Dynamic maceration; ** Soxhlet extraction; *** supercritical fluid extraction with CO_2_; ^1a^ hemp inflorescences; ^1b^ decarboxylated hemp inflorescences; ^2a^ hemp stalks; ^2b^ decarboxylated hemp stalks; ^3a^ hemp protein flour; ^3b^ decarboxylated hemp protein flour.

**Table 4:** Scavenging activity of the extracts towards ABTS and DPPH radicals expressed as IC_50_ (µg/mL). Trolox was used as a positive control. Values are the mean ± standard deviation of three different experiments conducted in triplicate.

	IC_50_ (µg/mL)	
	ABTS	DPPH
HS ^1^	125.1 ± 3.3	550.1 ± 4.5
DHS ^2^	87.2 ± 2.2	348.5 ± 2.4
HFP ^3^	>2000	>5000
DHFP ^4^	>2000	>5000
HI ^5^	85.3 ± 2.4	1122.0 ± 7.7
DHI ^6^	38.1 ± 1.1	535.7 ± 4.7
Trolox	15.9 ± 1.3	29.3 ± 1.5

^1^ Hemp stalk; ^2^ decarboxylated hemp stalk; ^3^ hemp proteic flour; ^4^ decarboxylated hemp proteic flour; ^5^ hemp inflorescences; ^6^ decarboxylated hemp inflorescences.

**Table 5:** Weak gel parameters for all samples at different temperatures.

T °C	20 °C		40 °C		60 °C	
ID	A [Pa·s]	z [-]	A [Pa·s]	z [-]	A [Pa·s]	z [-]
S1 *	354,241 ± 7000	7.5 ± 0.3	164,373 ± 900	8.4 ± 0.4	133,627 ± 7000	8.5 ± 0.5
SY1 **	305,042 ± 10,000	7.4 ± 0.1	176,235 ± 7000	8.0 ± 0.3	1,059,128 ± 1000	9.1 ± 0.1
SY2 **	297,758 ± 11,000	7.5 ± 0.4	124,496 ± 3000	6.7 ± 0.1	94,302 ± 2000	9.4 ± 0.6
SY3 **	245,606 ± 11,000	7.8 ± 0.4	114,326 ± 2000	7.5 ± 0.5	82,550 ± 5000	8.5 ± 0.8

* Base formulation without Greek yogurt. ** Base formulation with the addition of Greek yogurt at the percentages reported in Table 1.

**Table 6:** Color parameters of all snack bars after baking.

ID	L*	a*	b*	BI
S1 *	45.4 ± 1.7	2.5 ± 0.2	20.4 ± 0.4	42.8 ± 0.6
SY1 **	49.3 ± 0.6	3.1 ± 0.2	21.9 ± 0.9	43.7 ± 1.1
SY2 **	49.2 ± 1.5	3.2 ± 0.1	22.2 ± 0.2	44.6 ± 0.6
SY3 **	45.9 ± 0.1	3.6 ± 0.4	21.8 ± 0.6	46.8 ± 1.7

* Base formulation without Greek yogurt. ** Base formulation with the addition of Greek yogurt at the percentages reported in Table 1.

## Data Availability

The data used to support the findings of this study can be made available by the corresponding author upon request.
